# Common and differential genetically pathways between ulcerative colitis and colon adenocarcinoma 

**Published:** 2017

**Authors:** Somayeh Akbari, Mostafa Hosseini, Majid Rezaei-Tavirani, Mostafa Rezaei-Tavirani, Seyed Hamid Salehi, Mahdi Alemrajabi, Padina Vaseghi-Maghvan, Somayeh Jahani-Sherafat

**Affiliations:** 1 *Faculty of Medicine, Iran University of Medical Sciences, Tehran, Iran*; 2 *Proteomics Research Center, Shahid Beheshti University of Medical Sciences, Tehran, Iran*; 3 *Gastroenterology and Liver Diseases Research Center, Research Institute for Gastroenterology and Liver Diseases, Shahid Beheshti University of Medical Sciences, Tehran, Iran*

**Keywords:** Ulcerative colitis, Colon adenocarcinoma, PPI network, Cytoscape, Gene ontology

## Abstract

**Aim:**

In the present study, genes of Ulcerative Colitis and Colon Adenocarcinoma (COAC) were extracted by string App in Cytoscape software version 3.5.1. Then protein- protein interaction (PPI) networks analyzed.

**Background:**

One of the most common chronic digestive problems is ulcerative colitis (UC) especially in developing countries. Prevalence of the disease is reported about 7.6 to 245 cases per 100,000 per year. UC can lead to colon cancer that is the third malignancy related death in the world. So awareness of the future of the patient with UC and the possibility of colon cancer is a very helpful approach.

**Methods:**

The analysis was based on centralities values. The goal is determining common gene pathways and differential gene pathways of the two diseases.

**Results:**

Results showed there are 11 and 29 central genes related to COAC and UC respectively. At least five common key genes between the two diseases were introduced. The number of 26 terms related to the common key genes were determined and clustered in seven clusters.

**Conclusion:**

ALB, AKT1, TP53, SRC and MYC are the common genes that play crucial roles in the related biological processes of UC and COAC. Besides introducing the common genes the differentiate genes related to the two diseases were proposed.

## Introduction

 Colorectal cancer is known as the fourth commonest cancer in the world (1). It is a big problem in industrial countries however, its rate in developing countries is increased (2). Numbers of 49700 related death to colon cancer and 93090 new cases are reported in United States at 2015 (3). Survival of patients related to early diagnosis and since there is no proper and effective method, the mortality rate of colon cancer is high. Studies show people's lifestyle, such as nutrition and physical activity, are effective (4). In addition to, chronic digestive problems provide conditions for the onset of gastrointestinal cancers (5). Ulcerative Colitis (UC) and Crohn’s disease are the most common gastrointestinal inflammations. Many researches showed the connection between the two diseases (6). Investigations revealed that UC after 8-10 years increases significantly the risk of colorectal cancers (7). UC can affect rectum and colon, especially sigmoid colon and rectum are damaged parts in this disease. UC is common at any age and its main reason is unknown but aberrant activity of immune system, genetically factors, excessive and improper activity of colon bacteria or presence of some viruses and unpopular bacteria in gastrointestinal system were introduced as main risk factors of the disease (8). Diagnostic methods for UC are colonoscopy, blood test for finding out infection and inflammation factors and stool test for finding out blood cells in stool. Removing a part of colon is one of the complications of the disease that suggested to those who have UC for more than 8 years (9). Therefore, identifying the common and different genetic pathways of these two diseases can help to improve the lives of patients with UC. This way, can estimate the risk of colon cancer in people with UC. There are many experiments aimed at discovering the genetic similarities of these two diseases. So some useful protein and genetically data bases have been prepared valuable and extensive information about these two diseases (10, 11). Bioinformatic methods and protein-protein interaction network analysis can introduce common genetically pathways and differential biomarkers for UC and COAC (12, 13).

In this study, all the related genes to UC and COAC were extracted and PPI networks were analyzed that led to introduce differential biomarkers and common genetically pathways between the two diseases. 

## Methods

The related genes to UC and colon adenocarcinoma were from STRING App. of Cytoscape software version 3.5.1. The related PPI networks of the two diseases were constructed by Cytoscape software separately. The common genes between the two diseases were determined and in addition to the other related genes were included in a PPI network. The networks were analyzed and the central node (hub, bottleneck and hub-bottleneck nodes) were determined. Mean+2SD used as cut off value, to determine the hub-nodes (14). Five percent of top nodes based on betweeness value were selected as bottleneck nodes (15). The common nodes between hub-genes and bottleneck genes were identified as hub-bottleneck nodes (16). Since GO can provide useful information about roles of the genes (17), GO analysis of crucial genes was performed by ClueGO application in cytoscape software. Finally, the determined biological processes were clustered. Statistical significance were P-value≤0.01. 

## Results

**Figure 1:**
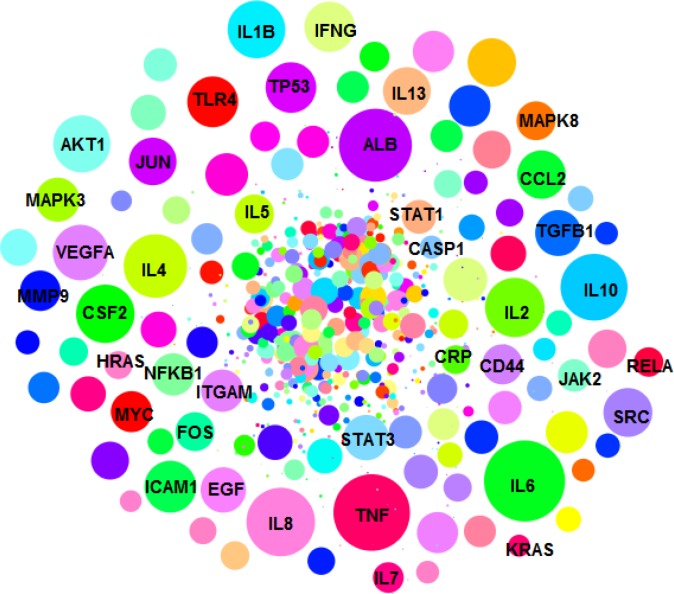
PPI network of UC including 843 genes is presented. The nodes are layout based on degree value (the bigger size is corresponded to more amount of degree value

**Figure 2.**
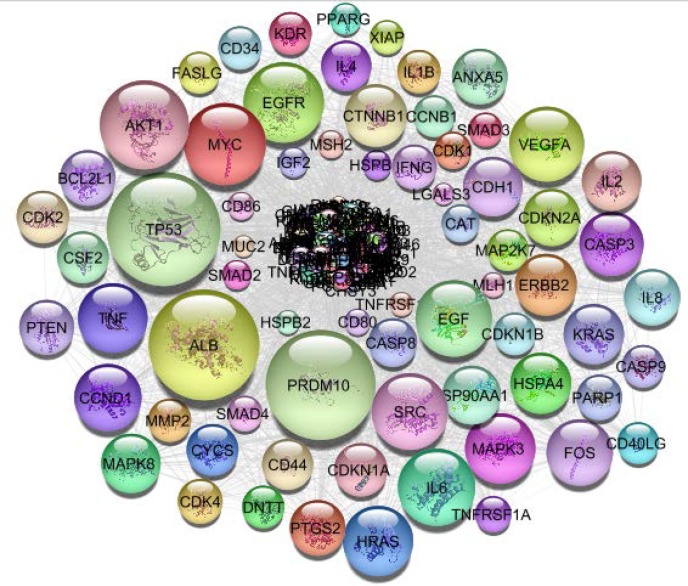
PPI network of colon adenocarcinoma including 376 genes is presented. The nodes are layout based on degree value (the bigger size is corresponded to more amount of degree value

The numbers of 843 and 376 related genes to UC and colon adenocarcinoma were extracted from string App. of Cytoscape software. The related networks were constructed and analyzed (Figures 1-2).

**Figure 3:**
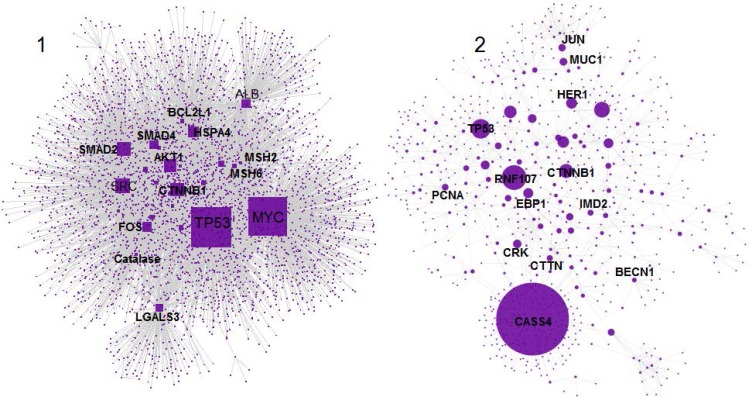
**A** PPI network including the common and the related genes between two diseases is presented. The nodes are organized in two connected components 1 and 2. The large and small connected components include 3581 and 809 nodes respectively. The nodes are layout based on degree value (the bigger size is corresponded to more amount of degree value

**Figure 4:**
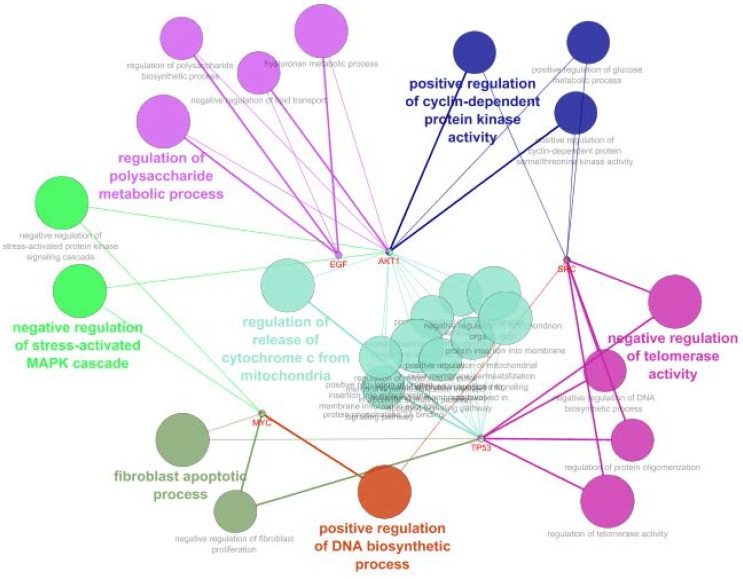
Schematic representation of GO analysis including biological pathway of common genes (the red color nodes) between UC and COAD.

More analysis referred to 65 common genes between the two groups of the related genes to the two types of diseases. A PPI network including these common genes and the related genes was constructed. 

**Table 1:** Hub-bottleneck nodes related to the common genes PPI network are presented

R	Genes	Description
1 2 3 4 5 6 7 8 9 10 11 12 13 14 15 16 17 18 19 20 21 22 23 24 25 26 27 28 29	TP53 MYC CTNNB1 SRC SMAD2 AKT1 HSPA4 SMAD4 FOS CASS1 ALB TNFRSF1A HRAS PPARG CDKN2A LGALS3 CCND1 CDH1 MSH2 KRAS VDR BCL2L1 MSH6 EGF SMAD7 CD44 VEGFA CDKN2A CAT	Cellular tumor antigen p53 Myc proto-oncogene protein Catenin beta-1 Proto-oncogene tyrosine-protein kinase Src Mothers against decapentaplegic homolog 2 RAC-alpha serine/threonine-protein kinase Heat shock 70 kDa protein 4 Mothers against decapentaplegic homolog 4 Proto-oncogene c-Fos Cas scaffolding protein family member 1 Serum albumin Tumor necrosis factor receptor superfamily member 1A GTPase HRas Peroxisome proliferator-activated receptor gamma Cyclin-dependent kinase inhibitor 2A Galectin-3 G1/S-specific cyclin-D1 Cadherin-1 DNA mismatch repair protein Msh2 GTPase KRas Vitamin D3 receptor Bcl-2-like protein 1 DNA mismatch repair protein Msh6 Pro-epidermal growth factor Mothers against decapentaplegic homolog 7 CD44 antigen Vascular endothelial growth factor A Cyclin-dependent kinase inhibitor 2A Catalase

As it is shown in figure 3, the network included 4390 nodes which are organized in the two main connected components. As it is depicted in figure 3, component-1 is a small sub network compared to component-2. Therefore, amounts of degree value in component-1 are smaller than the similar values in component-2. When the nodes of the two components analyzed together, the mean value of degree was smaller than the mean value of degree in the case of component-1. This point leads to lower cut off for degree value in the analysis of the nodes of the two components together relative to component-2. In the other hand, the top nodes of component-2 may be vanished. Due to avoiding of possible error the hub and bottleneck nodes for the common genes network were determined in the case of the two situations. 

**Table 2.** Hub-bottleneck nodes related to the two main connected components of common genes PPI network are presented

R	Hub-bottleneck genes of main connected component-1	Hub-bottleneck genes of main connected component-2
1 2 3 4 5 6 7 8 9 10 11 12 13 14 15 16 17 18	TP53 MYC CTNNB1 SRC SMAD2 AKT1 HSPA4 SMAD4 FOS ALB TNFRSF1A HRAS PPARG CDKN2A LGALS3 CDH1 CCND1 MSH2	TP53 SNW1 CTNNB1 CASS1

**Table 3:** Hub-bottleneck nodes related to the COAC PPI network are presented

Description	Gene	R
Serum albumin Cellular tumor antigen p53 PR domain zinc finger protein 10 RAC-alpha serine/threonine-protein kinase Catenin beta-1 epidermal growth factor receptor Myc proto-oncogene protein GTPase HRas Proto-oncogene tyrosine-protein kinase Src Mitogen-activated protein kinase 3 epidermal growth factor	ALB TP53 PRDM10 AKT1 CTNNB1 EGFR MYC HRAS SRC MAPK3 EGF	1 2 3 4 5 6 7 8 9 10 11

The determined hub-bottleneck nodes of the common genes network, the main connected components of the common genes network and the separated networks of the two diseases are presented in the tables 1-4 respectively. Based on early description and comparing tables 1 and 2, the hub-bottleneck nodes rows 20-29 in table 1 were excluded and SNW1 was added to the content of table 1. The final hub-bottleneck genes of common genes PPI network are shown in the table 5. Hub-bottleneck nodes of UC and COAC networks and the common hub-bottleneck genes between the two diseases are shown in table 6. For more details, see table 6. As it is shown in table 6 five genes including ALB, AKT1, TP53, SRC and MYC were the key genes in the common genes between UC and COAC. 

**Table 4:** Hub-bottleneck nodes related to the UC PPI network are presented.

Description	Gene	R
Serum albumin Interleukin-6 RAC-alpha serine/threonine-protein kinase Cellular tumor antigen p53 Tumor necrosis factor Proto-oncogene tyrosine-protein kinase Src Interleukin-8 Vascular endothelial growth factor A epidermal growth factor Mitogen-activated protein kinase 3 Interleukin-10 Myc proto-oncogene protein Interleukin-1 beta Interleukin-4 Toll-like receptor 4 Interleukin-2 Proto-oncogene c-Fos Transcription factor AP-1 Interleukin-13 Transforming growth factor beta-1 Granulocyte-macrophage colony-stimulating factor Intercellular adhesion molecule 1 Mitogen-activated protein kinase 8 Signal transducer and activator of transcription 3 Interferon gamma	ALB IL6 AKT1 TP53 TNF SRC IL8 VEGFA EGF MAPK3 IL10 MYC IL1B IL4 TLR4 IL2 FOS JUN IL13 TGFB1 CSF2 ICAM1 MAPK8 STAT3 IFNG	1 2 3 4 5 6 7 8 9 10 11 12 13 14 15 16 17 18 19 20 21 22 23 24 25

**Table 5:** Hub-bottleneck nodes related to the common genes PPI network are presented. Content of this table is provided by comparing tables 1 and 2

R	Genes	Description
1 2 3 4 5 6 7 8 9 10 11 12 13 14 15 16 17 18 19 20	TP53 MYC CTNNB1 SRC SMAD2 AKT1 HSPA4 SMAD4 FOS CASS1 ALB TNFRSF1A HRAS PPARG CDKN2A LGALS3 CCND1 CDH1 MSH2 SNW1	Cellular tumor antigen p53 Myc proto-oncogene protein Catenin beta-1 Proto-oncogene tyrosine-protein kinase Src Mothers against decapentaplegic homolog 2 RAC-alpha serine/threonine-protein kinase Heat shock 70 kDa protein 4 Mothers against decapentaplegic homolog 4 Proto-oncogene c-Fos Cas scaffolding protein family member 1 Serum albumin Tumor necrosis factor receptor superfamily member 1A GTPase HRas Peroxisome proliferator-activated receptor gamma, Cyclin-dependent kinase inhibitor 2A, Galectin-3 G1/S-specific cyclin-D1, Cadherin-1 DNA mismatch repair protein Msh2, SNW domain containing 1

**Figure 5:**
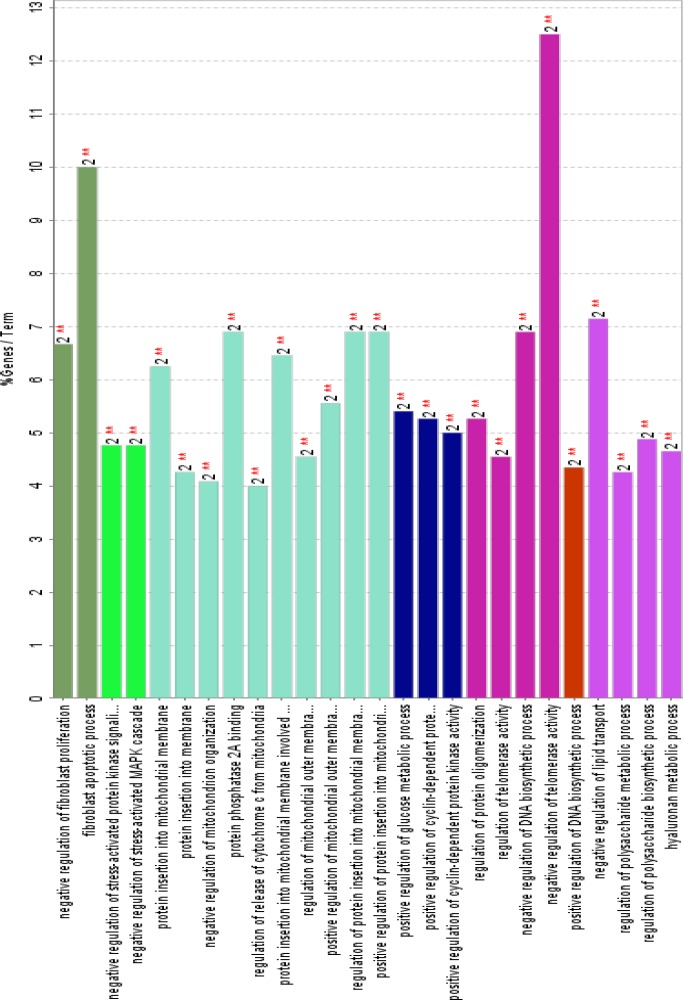
The related biological processes of common genes between UC and COAD. Inclusion criteria were at least two genes attribution in term and 4% gene/term. P-value for all terms were less than 0.01

**Figure 6.**
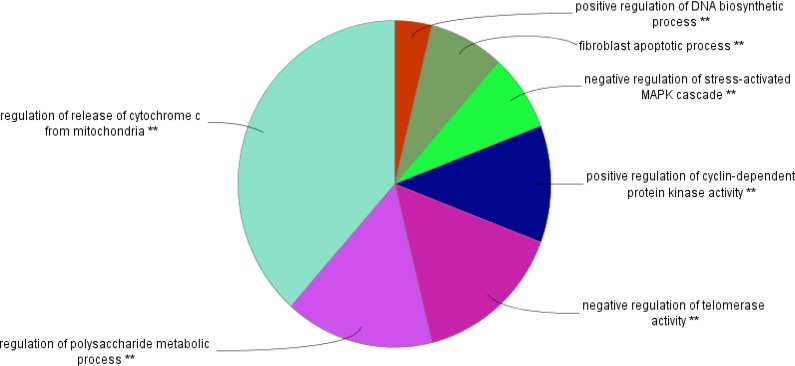
The related biological processes of common genes between UC and COAD are clustered in seven groups. P-value for all terms were less than0.01.

Gene ontology analysis of these key genes was performed by ClueGO application of Cytoscape (details were illustrated in figures4-6).

## Discussion

In many cases there are closed similarities between two or more diseases which may lead to misdiagnosis and ineffective treatment of patients (18). Precise diagnosis for such diseases implies complex and in the most condition aggressive tolls and methods. Colonoscopy and pathology evidences are the two well-known diagnostic tools for UC and COAC (19, 20). These approaches are valuable methods in the advanced stage of diseases but a noninvasive, differentially and effective tool especially for early stage detection of diseases always is required. Biomarkers are the specific and sensitive agents that find in the various parts of body and can be used as accurate diagnostic factors specially when are accessible in the serum (21). In the recent years suggestion of biomarker panels is attracted more attention in medicine (22, 23). Relationship between large numbers of genes and UC and COAC makes it possible that the two diseases be analyzed via PPI network approach. Construction of two scale free networks (12) provides the numbers of crucial genes which may be critical in diagnosis, differentiation and even treatment of the two diseases. 

Network analysis led to introduce 11 and 25 related central genes to COAC and UC respectively. Since the UC network is bigger than COAC network, it is logical that numbers of UC network key genes be more than the key genes of the other network. As it is depicted in table 3 all of the presented genes are well-known oncogenes which are related to the gastrointestinal cancers. In the other hand, the tabulated genes in table 4 include numbers of oncogenes and inflammatory proteins such as Ils. Since the introduced genes for the two diseases are corresponded to the previous studies, it seems that network approach is the right method for gene screening among large numbers of genes. When the content of tables 3 and 4 which correspond to the key genes of the two diseases were compared seven common critical nodes determined. ALB, TP53, AKT1, MYC, SRC, MAPK3 and EGF are the crucial genes in UC and COAC. In the other hand, network analysis of a constructed network of the 65 common genes (see table 5) led to introduce 20 critical common nodes between the two diseases. ALB, TP53, AKT1, MYC and SRC are five crucial common genes which were determined in the two analytic methods. ALB as an important carrier plays central roles in transferring of various types of ligands in body. Transferring broad spectra of drugs, metabolites and hormones is an essential role of albumin (24, 25). Expression change of ALB in large number of diseases is reported (26, 27). Since albumin is a house keeping gene (28), it is not a specific biomarker for UC or COAC. Expression change of TP53, AKT1, MYC, and SRC in numerous diseases especially cancers is confirmed (29-32). Relationship between TP53 and various kinds of cancers (almost all cancer diseases) is studied and discussed in the precise details (33, 34). However, we focused on common genes related to many cases of cancers but it is possible that the expression patterns as like amounts of expression and down or up regulation of these genes separately or in a combined panel be specific in relationship with a certain cancer. Since about 50% of related key genes of COAC network are presented in the UC it is corresponded to the closed correlation between the two diseases. It seems these introduced genes are a potent core to change UC to COAC. In the other hand, this closed similarity between two diseases may imply revision in treatment of UC. Probably therapeutic protocol of UC may resemble as COAC at least partially. Treatment of UC mainly is depended to 5-aminosalicylates, corticosteroids, and immunosuppressants, such as purine antimetabolites and cyclosporine (35). However, it is reported that 5-aminosalicylates may be effective in colorectal cancers prevention (36). The Basis of UC treatment is established on corticosteroids in combination with cyclosporine (37). 

As it is shown in table 6 seven types of interleukins are correlated to UC. These numbers of ILs are about 40% of the key genes that are presented in the UC network and are not included in the specific panel of COAC. Since UC is essentially related to the inflammatory system, this finding refers to power of PPI network analysis to discover new aspects of diseases.

GO analysis led to introduce 26 related terms to the five crucial common genes, which were clustered in seven groups. Since at least two key genes are involved in each term therefore, 40% central genes are presented in all terms. Positive regulation of DNA biosynthetic process is the smallest group including one term which is related to NYC and SRC genes. It is reported that SRC signaling cascade induces MYC expression and DNA synthesis (38). Regulation of release of cytochrome C from mitochondria is the largest cluster which is correlated to TP53 and AKT1 genes. Regulation of release of cytochrome C from mitochondria is a part of regulation of BCL2 of apoptosis (39). Based on figure 4; AKT1, TP53, SRC, MYC and EGF are involved in four, three, three, three and two clusters respectively. AKT1 is related to 19 terms among 26 terms. This wide participation (it is involved in 73% of total terms) indicates the important role of AKT1 in both diseases. In addition to the role of AK1 in apoptosis and human cancers; its participation in other diseases and disorders such as schizophrenia is reported and discussed in details (40, 41). 

It can be concluded that there is a main similarity between UC and COAC which implies revision of therapeutic aspects of UC. It may be application of mild anticancer drugs for treatment of UC added to corticosteroids. The differential elements between the two studied diseases may be useful in diagnostic features of UC and COAC.
